# rTMS of the superior parietal lobule improves contrast discrimination but has no effect on the perception of distance between stimuli in the image plane

**DOI:** 10.1177/03010066221114571

**Published:** 2022-08-15

**Authors:** Nicolo Biagi, Charlotte Goodwin, David T. Field

**Affiliations:** 6816 University of Reading, UK

**Keywords:** TMS, visual separation, attention, tracking/shifting attention, frontal eye field

## Abstract

The superior parietal lobule (SPL) is a region of the brain that has been associated with a diverse range of high-level visual and cognitive functions. This suggested the possibility that it supports a lower-level function that is engaged by a wide range of experimental tasks. Analysis of tasks used in previous studies suggests that one such lower-level function might be the perception of the distance between stimuli in the image plane. In this study, we applied online high-frequency repetitive transcranial magnetic stimulation (rTMS) over the left SPL or the vertex in order to further investigate the role played by this region in the perceived visual separation between points. As a control task, we asked participants to detect the difference in contrast between two Gabor patches. The results failed to support the main hypothesis, but we unexpectedly found that rTMS to left SPL improved peripheral contrast discrimination. Previous studies have found that rTMS to the right frontal eye field, which has strong functional connectivity with the SPL, has the same effect, suggesting the two areas work together to influence early visual areas.

## Introduction

The brain areas in the occipital lobe that are relevant for the visual system mostly contains neurons whose visual receptive fields are tuned to specific retinal locations and at the population level these neurons are organized into retinotopic maps. This system is well suited to encode the positions of stimuli in a retinal coordinate frame. However, it does not provide in any direct way information about the separations (retinal distance) between individual stimuli. Specifically, neurons have not been found in visual cortex whose firing rate depends on the separation or distance between two stimuli. Nonetheless, humans are good at perceiving the separation between two points in visual space when it defines such properties such as the width of a circle (Morgan, 2005) or the height of a rectangle (Nachmias, 2008).

One suggested explanation of this perceptual ability is that a higher visual area, such as that previously proposed to be responsible for magnitude perception (Bueti & Walsh, 2009; Walsh, 2003) reads out position information about edges or corners of salient and attended stimuli from early visual areas and computes separations between positions (Harvey et al., 2015; Schwarzkopf, 2015). Consistent with this proposal, the precision of psychophysical judgements of higher order properties of shape such as geometrical angle and aspect ratio is good and cannot be accounted for by sensitivity to properties of the components of the shapes (Chen & Levi, 1996; Heeley & Buchanan-smith, 1996; Nachmias, 2008). The computational mechanism by which this is done is unknown but is likely to be different from the kind of formal trigonometry that a computer vision algorithm might use to solve this problem. Here, rather than focusing on the computational mechanism we aim to determine which brain area performs the read-out and the computation. Note that our investigation focuses on the perceived visual separation between points in the coordinate frame of the retinal image, not the perceived distance or separation in depth between objects in the world.

The superior parietal lobule (SPL) is a part of the parietal lobe, located in the posterior part of the brain, close to the midline. Brain imaging studies have associated many different functions with this region and report similar activation coordinates in SPL for different functions, for example, shifting spatial attention between locations (Vandenberghe et al., 2001); the perception of heading direction (Peuskens et al., 2001); the perception and planning of the path of travel during locomotion (Billington et al., 2010; Field et al., 2007); and motion tracking under attentional load (Jovicich et al., 2001). One study set out to study activation produced in SPL by making smooth pursuit eye movements, but instead found that activation in the region appeared to be driven by the presence of perceived relative motion between display elements (Ohlendorf et al., 2010).

Whilst the authors of these studies reported contrasting explanations for SPL activation that reflected their particular sets of stimuli and tasks it is possible that a single underlying function could provide a unifying explanation of the activation in these apparently diverse studies. The experimental tasks used in all but one of the aforementioned studies would require participants to shift their attention between elements of the visual display, which suggests that shifting spatial attention may be the underlying function explaining these results, as proposed by Vandenberghe et al. (2001). On the other hand, all these studies – including Vandenberghe’s – also used stimuli in which the visual percept is that of changing visual separations between stimulus elements. Therefore, an alternative possibility is that SPL supports the perception of visual separation, which is why it was selectively activated in all the studies reviewed here. One exception is the study of Ohlendorf et al. (2010), in which the pattern of results considered in relation to the stimuli used does not appear to implicate SPL in attention shifting, but is consistent with a role in the perception of visual separation.

As a step towards determining whether either of the two basic functions described above might explain the selective activation of SPL by a range of experimental tasks, we directly tested the attention shifting hypothesis of SPL in an fMRI experiment and found that it was unable to account for the results (Field & Goodwin, 2016; Goodwin, 2021). Specifically, when a single target square displaces in an otherwise featureless environment and the displacement is tracked by saccadic eye movements SPL activation is very low, despite the mandatory shift of spatial attention to the new target location that occurs before each saccadic eye movement (Deubel & Schneider, 1996). Yet when a task irrelevant central cross was added to the display and the participant continued, as before, to make saccadic eye movements to track the displacing square strong activation occurred in SPL; adding the task irrelevant cross changed nothing in terms of saccade related spatial attention shifting, but it did introduce the percept of time-varying visual separation to the display which we propose drives activation in the SPL subregion. This result is problematic for the attention shifting hypothesis of SPL activation, which would have to make the implausible claim that saccades to targets can be made without shifting spatial attention in order to explain the results, but consistent with the proposal that a subregion of SPL processes visual separations.

**Figure 1.**
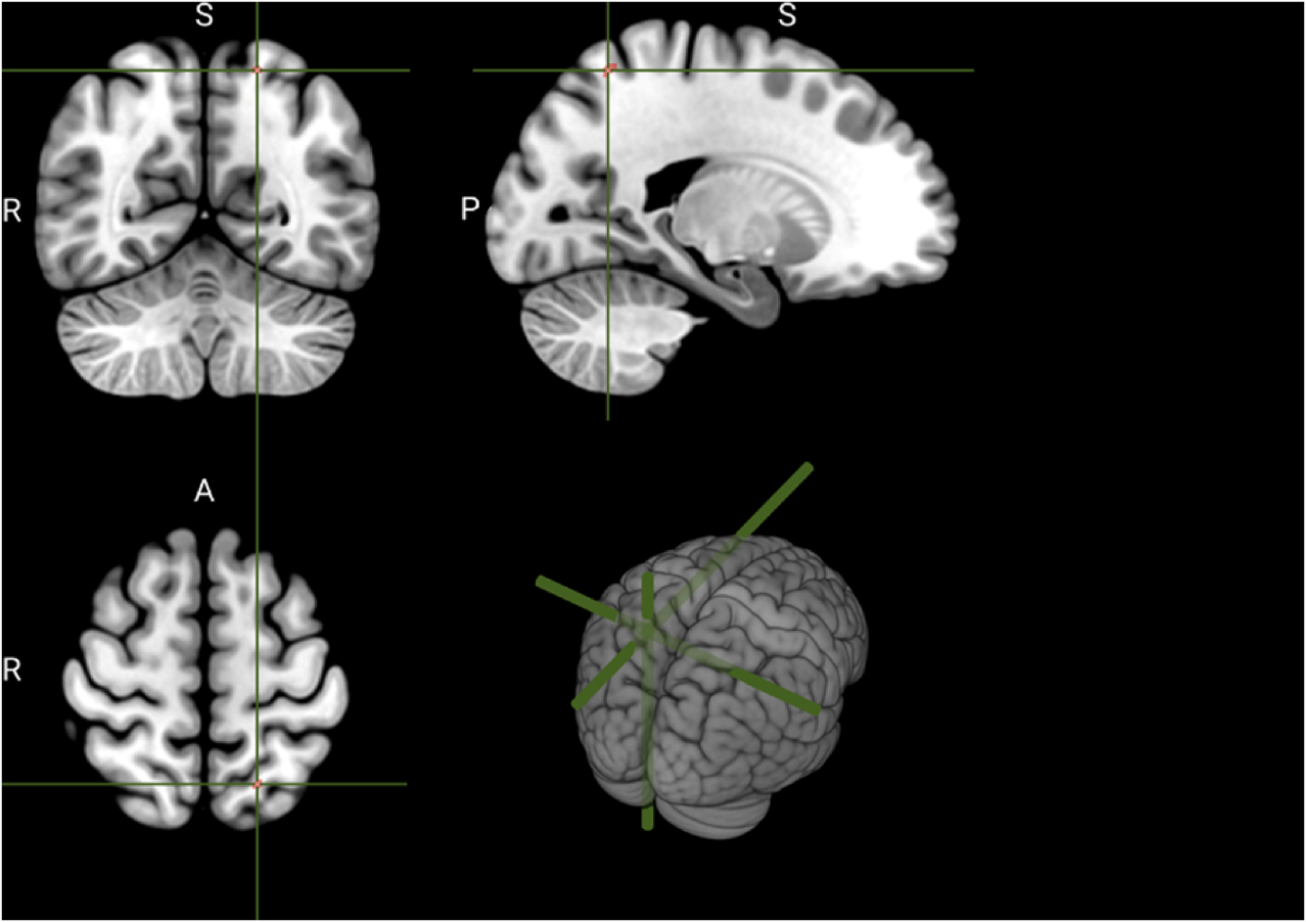
The MNI coordinates in the SPL targeted by TMS stimulation.

The present study aimed to test the proposal that the subregion of SPL shown in Figure 1 was critical for the processing and perception of visual separation using a non-invasive brain stimulation technique known as transcranial magnetic stimulation (TMS). TMS can disrupt targeted brain regions to reveal their causal role in task performance. The behavioural task performed while TMS was applied to SPL was a psychophysical visual separation judgment task, in which two different pairs of dots were presented on a computer screen and the participant indicated in which pair the distance between the dots was larger. We predicted that TMS to the SPL would result in less precise performance on this task but did not expect accuracy to be affected by TMS to SPL. In the experiment the stimuli were confined to the right visual field and the TMS was applied to SPL in the contralateral hemisphere. This arrangement followed from the fact that SPL is found bilaterally in the brain and shows a bias to process the contralateral side of visual space, that is, the left visual field was processed mainly in the right hemisphere of SPL (Silver & Kastner, 2009). To increase methodological rigor, we also applied TMS to a control region (i.e. the vertex) that was not thought to play a role in processing visual separation. For the same reason, we included a control psychophysical task that did not require judgment of visual spatial separation but shared many of the generic task features, such as deciding between two alternatives and pressing a button, with the main task of interest. Our first prediction was that the slopes of the psychometric functions obtained during the visual separation task would be shallower when TMS stimulation was delivered over SPL compared to when it was delivered over the vertex. But for the hypothesis that the SPL subregion we targeted is the specific part of the brain that supported the perception of visual separation to be supported by this study an additional prediction must be fulfilled: that TMS delivered over SPL does not influence slopes of psychometric functions obtained from the control task. We had no specific reason to predict that TMS would differentially affect the point of subjective equality (PSE) in either the experimental or control task, and so performed an exploratory analysis of this.

## Method

### Participants

For this study 20 healthy participants (16 females, 4 males) were recruited for a two non-consecutive days TMS study at the University of Reading. This sample size was sufficient to detect an effect size of *d* = 0.57 for our one tailed prediction (power 0.8, alpha 0.05, paired samples *t*-test). Participants were recruited via the University of Reading Student Volunteer Panel (SONA), where the study was advertised. The age range of the participants varied from 19 to 28 years old (Median 21, range 19–28). All participants were informed that their participation in this study was voluntary and that they could withdraw at any time without providing a reason.

### Ethical Approval

This study was granted ethical approval by the University of Reading Ethics Committee (UREC) 17/24, expiration 1/10/2020. Due to the seizure-potential that TMS stimulation carries (Wassermann & Lisanby, 2001), participants were asked to complete a TMS screening form before each TMS stimulation. The TMS screening form was approved by UREC and was composed of 24 questions aimed to investigate if the participant had previous psychiatric, neurological, or other medical condition, and therefore was not eligible for the TMS stimulation (Rossi et al., 2009). Moreover, the experimental design took into account the TMS safety parameter specified by Wasserman et al. (2001) and Rossi et al. (2009) that was computed from the combined duration, intensity, and frequency of stimulation. Before the start of the TMS stimulation participants were reminded that they could withdraw at any time from the study without providing a reason.

### Apparatus and Materials

All the experiments presented in this study were programmed using Psychtoolbox (Brainard, 1997; Kleiner et al., 2007; Pelli, 1997), a freely available package toolbox for MATLAB. All the stimuli were displayed on a 24-inch ViewPixx monitor (1920 (V) × 1080 (H) pixels), placed 90 cm away from the participant. In order to reduce the head movements, participants were asked to rest their chin on a chinrest for the entire duration of the experiment (the chinrest was placed 90 cm away from the ViewPixx monitor).

### Design and Procedure

The design of this study was fully repeated measures, with every participant undergoing online TMS stimulation in each experimental condition over two different regions: the SPL and the vertex (control region). Each region was stimulated on a different day and there was at least a 48 h interval between the two sessions. For half of the participants, on Day 1 the stimulation was delivered over the SPL, and on Day 2 it was delivered over the vertex, while for the other half of the participants the order was reversed. In each session, both the control task and then experimental task were performed. For half of the participants the experimental task was presented first on both days, while for the other half the order of presentation was reversed.

In the experimental task, the effect of TMS on the perceptual judgment of visual distances was investigated. In order to do so, the PSE between two simultaneously presented visual separations was measured. This was done by presenting a two alternative forced choice task.

**Figure 2.**
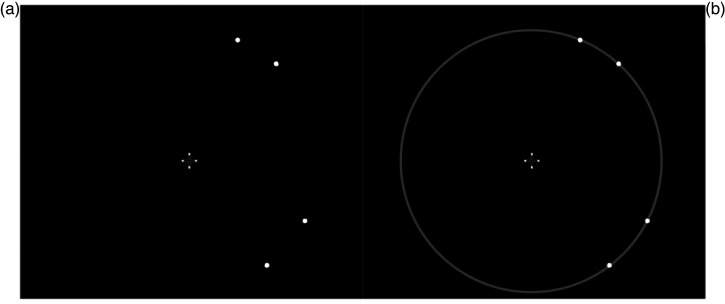
Stimuli used in the experimental task: (a) stimuli presented to the participants were a pair of dots presented above the fixation cross (2nd quadrant) and a pair presented below the fixation (4th quadrant); (b) dots making up the stimuli lay on an imaginary circle of radius 5 degrees of visual angle.

On each trial the participant was briefly presented with two pairs of white dots, and judged which pair defined the larger visual distance (see Figure 2). The TMS stimulation was paired with the brief presentation of the two set of dots.

**Figure 3.**
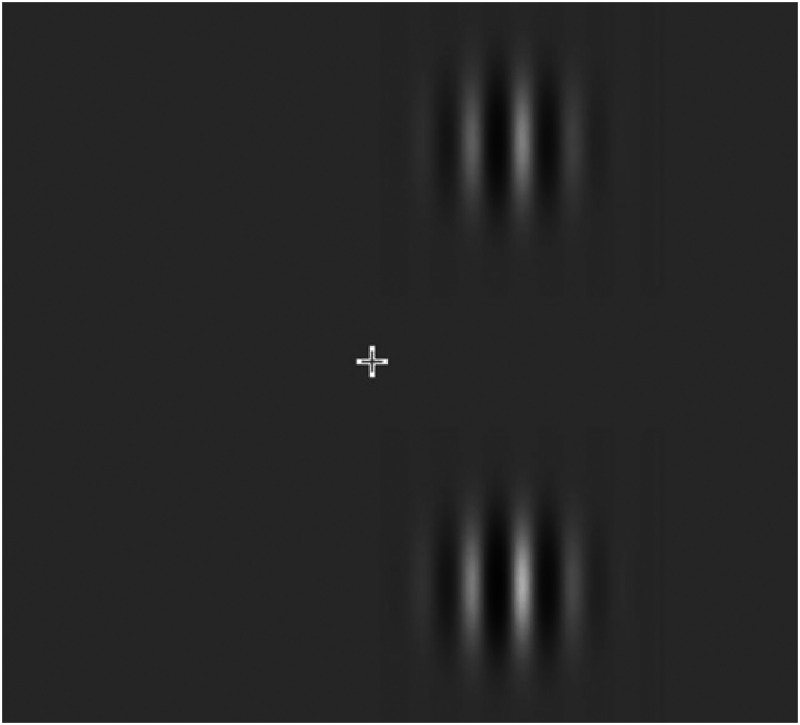
Stimuli used for the control task.

In the control experiment, the effect of TMS on the PSE between the contrast of two Gabor patches was determined (see Figure 3). The cognitive and motor aspects of this task were identical to those in the experimental task, but the perceptual comparison required did not involve spatial extent.

#### Stimuli

In the experimental task, two pairs of white dots and a fixation cross were presented against a black background (Figure 2a).

One set of dots was presented below the fixation cross (4th quadrant of the screen, using the fixation/centre of the imaginary circle as the origin), while the other set was presented above the fixation cross (2nd quadrant of the screen). All the individual dots lay on an imaginary circle with a radius of 5 degrees of visual angle (DOVA) that was centred on the fixation cross (Figure 2b). All the dots presented subtended 0.2 DOVA. From trial to trial, the visual distance between the two dots making up each pair was manipulated. The pair of dots presented below the fixation cross was defined as the ‘Standard’ and the distance between the two dots varied randomly from trial to trial between 2.59 and 5 DOVA. The location of the two dots making up the ‘Standard’ was randomly jittered within the quadrant by MATLAB on each trial.

The pair of dots presented above the fixation cross was defined as the ‘Comparison’ and the distance between these two dots in each trial was a percentage of the Standard. These percentages were 70%, 79%, 88%, 97%, 102%, 112%, 121%, and 130%, and each percentage was presented 30 times during the experiment. On each trial, the participant indicated whether the visual separation defined by the Standard, or the Comparison appeared larger using the up and down arrow keys on the keyboard.

The two pairs of dots were presented on the screen for only 200 ms to prevent saccadic eye movements during the trial, and participants were not allowed to look directly at them, they had to fixate at the centre of the screen (where a fixation cross was presented) and use their peripheral vision to detect them and complete the task. The fixation cross was composed of a black cross placed on top of a white one.

Each arm of the white fixation cross was set to 0.3 DOVA, while each arm of the black fixation cross was set to 0.2 DOVA. The line width of the white fixation cross was set to 0.2 DOVA, while the line width of the black cross was set to 0.1 DOVA.

In the control task, a fixation cross and two Gabor patches were presented against a grey background (Figure 3).

Both Gabor patches were presented to the right of the fixation cross, one above and the other one below it. The centre of both Gabor patches lay on the same imaginary circle that was used in the experimental task, which was centred on the fixation cross with a radius of 5 DOVA.

The fixation cross was composed of a grey cross placed on top of a white one. Each arm of the white fixation cross was set to 0.3 DOVA, while each arm of the grey fixation cross was set to 0.2 DOVA. The line width of the white fixation cross was set to 0.2 DOVA, while the line width of the grey cross was set to 0.1 DOVA.

On each trial the contrast of the Standard Gabor patch presented below the fixation cross was randomly selected between a range varying from 0.4 to 0.7 in steps of 0.1. The contrast of the Comparison Gabor patch presented above the fixation cross was a percentage of the contrast used of the Gabor below the fixation cross. During the entire experiment, eight different values were used as percentages (70%, 79%, 88%, 97%, 103%, 112%, 121%, and 130%), and each of them was presented 30 times. Both the Standard Gabor patch and the Comparison Gabor patch had a spatial frequency of 1 cycle per degree, were oriented vertically, had radius of 3°, and the sigma of the Gaussian envelope was 0.43°. The two Gabor patches were displayed on the screen for 200 ms.

#### Resting Motor Threshold

After the participant successfully completed the screening form and after obtaining written consent form, the resting motor threshold (RMT) was acquired on each day of the experiment for all the participants.

The RMT is the lowest intensity of stimulation needed to be delivered to the primary motor hand area (M1-HAND) in order to evoke a peak-to-peak motor evoked potential of 50 μV in at least five out of ten consecutive trials in the contralateral relaxed first dorsal interosseus muscle (Quartarone et al., 2005).

In order to define the starting position for the search for the M1-HAND area, the TMS coil was firstly placed on top of the vertex (defined as the mid-distance between the nasion-inion, and the left-right auricular bones) and then moved 1 cm to the left, away from the vertex and 4–5 cm forward (Groppa et al., 2012).

During the entire RMT assessment, the handle of the coil was pointed backwards at a 45° angle away from the midline, approximately perpendicular to the line of the central sulcus. For each subject, the RMT was determined as the intensity at which single pulses applied over the hand area of right M1 produced a visible muscle twitch in five of ten consecutive trials, which is a standard procedure in the field (Feredoes et al., 2006; Schutter & van Honk, 2006).

Once the RMT for the day was defined, we set the intensity of stimulation for the experimental tasks to 110% of that value. Mean  ±  SE RMT was 59.85  ±  1.5% maximum stimulator output (MSO) for the SPL and 59.65 ± 1.9 MSO for the vertex. Mean  ±  SE experimental stimulation intensity was 65.8  ±  1.7% MSO for the SPL and 66 ± 2.1% MSO for the vertex.

#### Location of the TMS Target

After defining the RMT and the intensity of TMS stimulation for the day, we located the target for the stimulation on that day. On the day in which the vertex was the target of the stimulation, the target was located in each participant as the mid-distance between the nasion-inion, and the left-right auricular bones. On the day in which the SPL was the target, the location was found using the Brainsight software (Brainsight TMS, Rogue Resolutions Ltd) and MNI coordinates. SPL is a large brain region, and so to target the most appropriate sub-region of SPL the MNI coordinates for TMS were selected on the same basis as in a series of fMRI studies running concurrently in the lab (Field & Goodwin, 2016; Goodwin, 2021). This involved taking the average of the peak *X*, *Y*, and *Z* coordinates of the SPL functional activations reported in the studies reviewed in the Introduction (Billington et al., 2010; Field et al., 2007; Jovicich et al., 2001; Ohlendorf et al., 2010; Peuskens et al., 2001; Vandenberghe et al., 2001). The resulting coordinates used for targeting TMS in the SPL were *x* = −20, *y* = −60, *z* = 60 (see Figure 1). Unfortunately, due to a major upgrade causing the MRI scanner to become unavailable, only nine participants had a T1w image that we could use to locate the SPL, so for the remaining participants we used a standardised 2 mm T1w that comes with the Brainsight software. The procedure for locating the SPL was the same in all the participants: after loading either the participant’s T1w image or the standardised 2 mm T1w included in Brainsight, the participant was asked to sit in front of the Polaris camera and wear a subject tracker, which was strapped to their forehead. Then the researcher used a pointer to point at the nasion, auricular bone on both the left and the right, in order to register the participant’s head within the Brainsight Software and recreate a skull based on the participant’s landmarks. After that the above MNI coordinates for the SPL subregion were entered, or for the vertex the landmark defined during the RMT procedure was used. After the TMS target for the day was located, participants were asked to place their chin on a chinrest, placed 90 cm away from a ViewPixx monitor, and then the TMS coil was placed over the target, and it was hold in place using a mechanical arm.

#### TMS Stimulation

The experimental and control tasks were both composed of 240 trials and during each trial a pattern of TMS pulses was delivered. During the experimental task the TMS stimulation was synchronised with the 200 ms presentation of the two sets of dots, while in the control task the TMS stimulation was synchronised with the presentation of the two Gabor patches. For both tasks the end of the TMS stimulation was paired with the removal of the stimuli from the screen.

**Figure 4.**
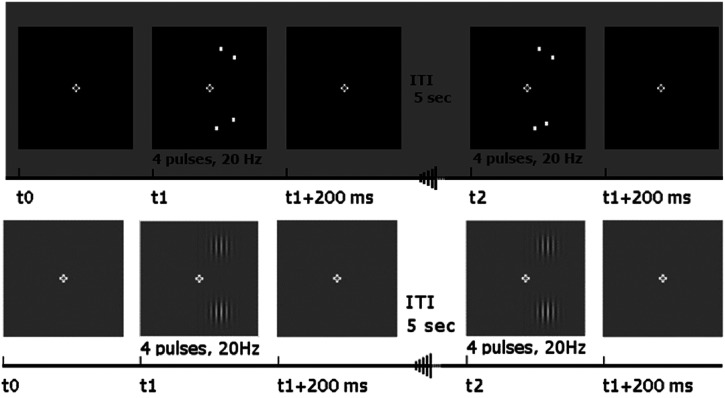
Timelines of the visual separation and contrast tasks. In the visual separation task (top panel) t0 is when the fixation cross is presented on the screen; at t1 the two pairs of dots are presented on the screen, one pair above and the other one below the fixation cross. The time interval between t0 and t1 was randomly jittered around 1000 ms, with minimum and maximum values of 750 and 1250 ms. The presentation of the stimuli was paired with the TMS pulses; after 200 ms the TMS stimulation stopped and also the stimuli were removed from the screen. The contrast task (bottom panel) was identical except that the pairs of dots were replaced by Gabor patches. In the visual separation task, participants indicated which of the two dot pairs defined a larger gap, while in the contrast task they indicated which Gabor patch had higher contrast.

Four pulses were delivered during a 200 ms time window (20 Hz) and the intensity of stimulation was set to 110% of the RMT acquired earlier that day; the pulses were delivered using a figure-of-8 coil, which was attached to a PowerMag machine (Mag & More GmbH, München, Germany). A 5 s ITI was inserted between each experimental trial, in order to avoid any add-up effects of the TMS (Hamidi et al., 2011). These timings are illustrated in Figure 4. Overall, in both the experimental and control conditions 960 pulses were delivered to each participant, 1920 in total on each day.

## Results

All the participants successfully completed both sessions of this study, and no data was discarded or excluded.

**Figure 5.**
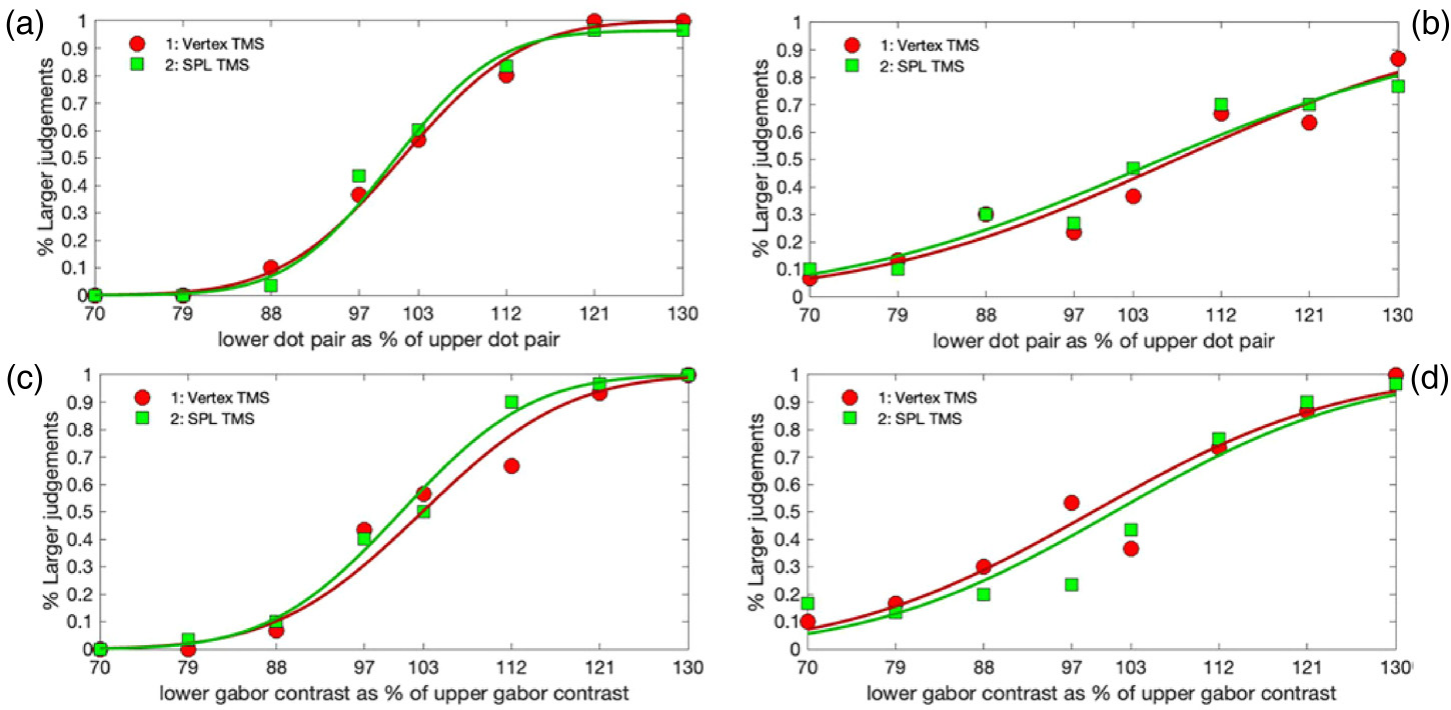
Four example cumulative normal functions from individual participants. (a) An example from a participant with relatively good performance in the visual separation task, reflected in a steep slope; (b) an example of a participant with relatively poor performance in the visual separation task; (c) and (d) provide similar examples of good and poor performance in the contrast judgement task.

By using the Palamedes toolbox for MATLAB (Prins & Kingdom, 2018), we fitted a cumulative normal function to the data acquired for each task on both days, resulting in four cumulative normal functions for each participant (Figure 5).

From each fitted psychometric function, we extracted and statistically analysed the PSE (i.e. the Comparison stimulus as a percentage of the Standard for the point where the two visual extents were judged to be equal) and the slope. The descriptive statistics of these two parameters are included in Table 1.

**Table 1.** Descriptive statistics for the visual separation task and the control task.

Parameter	Statistic	VisSep-SPL	VisSep-Vertex	Control-SPL	Control-Vertex
PSE	Mean	102.3	101.4	102.9	104.6
	Median	102.6	102.2	101.5	104.5
	SD	6.8	8.1	6.1	5.2
	SE	1.5	1.8	1.4	1.2
	Min	89.9	86.01	91.7	95.2
	Max	116.4	115.1	115.6	114.3
SLOPE	Mean	0.058	0.070	0.060	0.059
	Median	0.057	0.071	0.055	0.059
	SD	0.018	0.018	0.020	0.015
	SE	0.004	0.004	0.004	0.003
	Min	0.032	0.046	0.040	0.032
	Max	0.103	0.114	0.118	0.085

**Figure 6.**
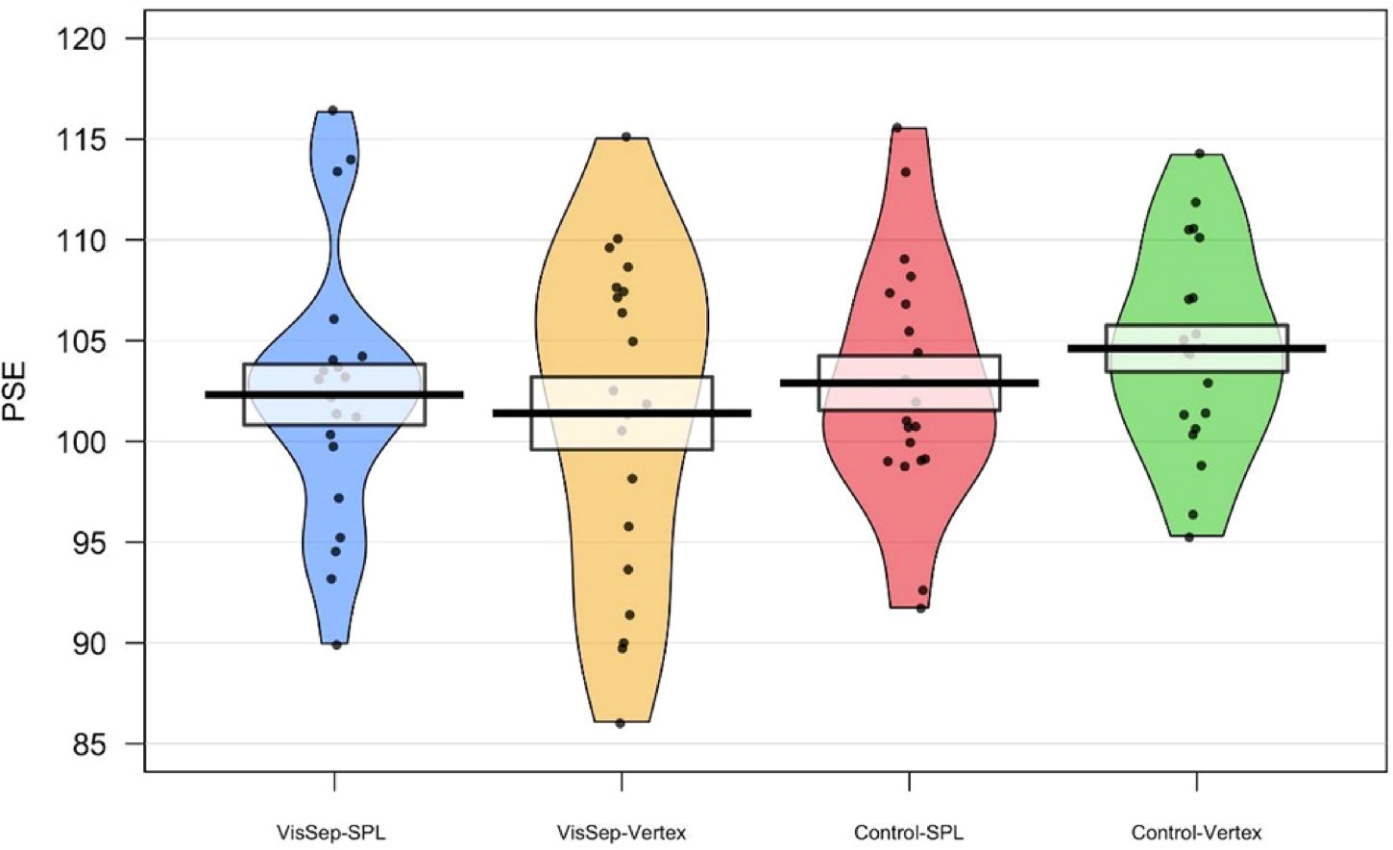
Pirate plots showing the mean, SD and distribution of the PSE for both the visual separation judgment and the control contrast judgment task, with TMS applied to either the SPL or the vertex.

**Figure 7.**
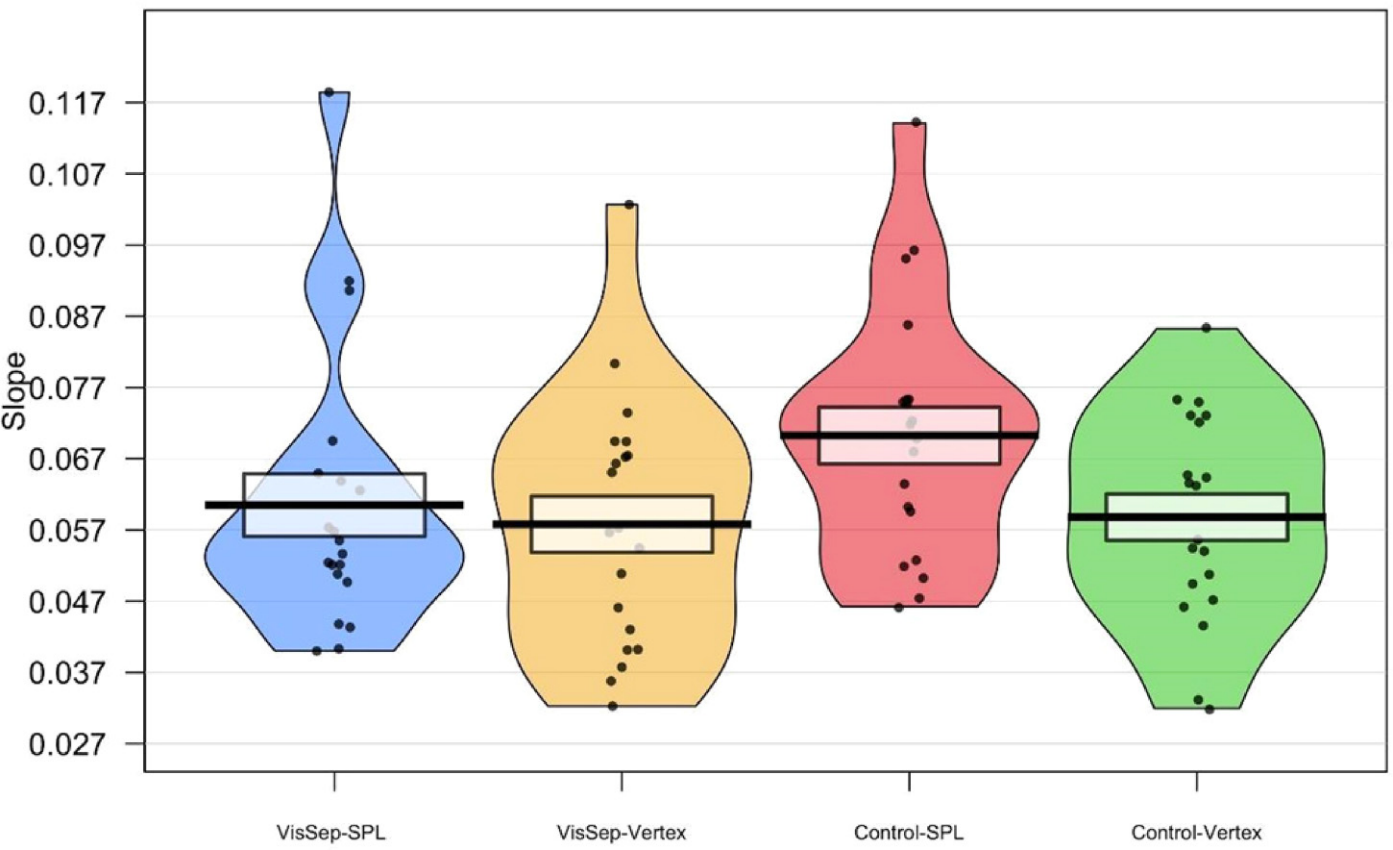
Pirate plots showing the mean, SD and distribution of the slope of the fitted psychometric functions for both the visual separation judgment and the control task, with TMS applied to either the SPL or the vertex.

Pirate plots showing the mean, SD, and distribution of the PSE and the slope in the four experimental conditions are presented in Figures 6 and 7.

### Influence of SPL TMS Compared to Vertex TMS on Precision of Visual Separation Judgments

Our prediction was that the TMS stimulation of the SPL should have affected the precision of the visual separation task. Moreover, we predicted that the disruptive effect of the TMS stimulation of the SPL should have resulted in a shallower slope for the psychometric function, compared to the slope obtained in the psychometric function for the same task when the stimulation was delivered over the vertex.

In order to test our prediction, a paired sample *t*-test was run for the slopes of psychometric function obtained from the visual separation tasks. There was not a significant difference between the slope obtained for the SPL stimulation (*M* = 0.061, SD = 0.020) and the slope obtained for the vertex stimulation (*M* = 0.058, SD = 0.018); *t*(19) = −1.046, *p* = .309; *d* = −0.23.

As an additional test of the prediction, we also calculated and analysed Weber fractions for each participant (Weber fraction = JND/PSE), which may provide a more sensitive index because they take into account the individual differences in PSE across the sample. A paired *t*-test was performed Weber fractions, and revealed no significant difference between SPL stimulation (*M* = 0.122, SD = 0.032) and vertex stimulation (*M* = 0.129, SD = 0.041); *t*(19) = −1.338, *p* = .197, *d* = −0.3.

### Influence of SPL TMS Compared to Vertex TMS on Precision of Judgments in the Control Contrast Discrimination Task

Our hypothesis suggested that the TMS stimulation would have no effect on the slope obtained in the control task.

A paired-sample *t*-test was run for the slopes of psychometric function obtained from the control task. Unexpectedly, the SPL stimulation resulted in steeper psychometric functions (more precise judgment) than the vertex stimulation, and this difference was significant (SPL *M* = 0.07, SD = 0.018; vertex: *M* = 0.059, SD = 0.015), *t*(19) = −3.322, *p* = .004; *d* = −0.74.

To further explore and confirm this unexpected result we also calculated and analysed Weber fractions for each participant (Weber fraction = JND/PSE). A paired *t*-test was performed on the Weber fractions, and this revealed a significant difference between SPL stimulation (*M* = 0.102, SD = 0.026) and vertex stimulation (*M* = 0.119, SD = 0.034); *t*(19) = −2.247, *p* = .037, *d* = −0.5.

### Exploratory Analysis of the Effect of TMS on the PSE (Bias)

We had no specific reason to predict that TMS would differentially affect the PSE in either the experimental or control task, and so performed an exploratory analysis of this. A paired sample *t*-test was run on the PSEs of the psychometric functions obtained from the visual separation task. There was not a significant difference between the PSEs obtained for the SPL stimulation (*M* = 102.3211, SD = 6.7698) and the PSEs obtained for the vertex stimulation (*M* = 101.3948, SD = 8.0682), *t*(19) = −0.893, *p* = .383; *d* = −0.2.

Another paired sample *t*-test was run for the PSEs of psychometric function obtained from the control task. There was not a significant difference between the PSEs of the psychometric function obtained for the control task when the TMS was delivered over the SPL (*M* = 102.892, SD = 6.047) and the PSEs obtained for the control task when the TMS was delivered over the vertex (*M* = 104.612, SD = 5.168), *t*(19) = 1.372, *p* = .186; *d* = 0.31.

## Discussion

The main purpose of this experiment was to test the hypothesis that part of the SPL is involved in supporting the perception of the 2D visual separation between two points. In order to achieve this goal, a 2-day TMS experiment was carried out. On the first day of the experiment the TMS stimulation was delivered over the left SPL while two different tasks were presented: the experimental task was aimed to measure the just noticeable difference in visual separation between two points. On the second day of the experiment the TMS was delivered over the vertex (a control site for TMS stimulation), while the same two tasks were presented. We predicted that TMS over SPL would reduce the precision of judgments of visual separation compared to TMS over the vertex, but not in a control task. We found no effect of SPL TMS on the precision of judgments of visual separation, but we unexpectedly found that TMS over SPL compared to vertex increased the precision of performance in the control task, which measured the ability to detect the difference in luminance contrast between two peripherally presented Gabor patches.

The results provide evidence against the hypothesis that the part of SPL we targeted with TMS plays a specific role in the ability to perceive visual separation. Although this interpretation depends upon a null effect of TMS to the SPL, it is strengthened by the fact we were able to detect a clear effect of SPL TMS on the control task; this demonstrates that the experiment was adequate from a technical viewpoint and could, in principle, have detected the predicted effect. Note that the experiment would have been highly unlikely to detect a potential effect of SPL TMS on bias, for example, perceiving visual separations as smaller due to TMS, because the Standard and Comparison stimulus were both presented at the same time as the TMS pulses were delivered and would both be influenced in the same way by it (Anstis, 2002; Rock, 1966). Analysis of the PSE was presented here for completeness.

We chose contrast discrimination as a control task because we assumed it was supported by early visual areas, but we unexpectedly found that precision of judgement in the control contrast sensitivity task was better following SPL TMS than following vertex TMS. As well as being unpredicted, the result is unusual in that delivering high-frequency repetitive transcranial magnetic stimulation (HF-rTMS) usually results in reduced rather than improved task performance (Rotenberg et al., 2014). Nonetheless, we believe the finding is genuine rather than Type 1 error because the effect size was medium/large, and when we performed a Bayesian paired samples *t*-test to follow this up the Bayes factor was 17.4, which indicates strong evidence. Furthermore, the finding is consistent with previous literature; similar effects have been observed for TMS to the frontal eye field (FEF) and very strong functional connectivity has been found between SPL and FEF; in a study of the resting state functional connectivity of the human FEF the strongest two foci were found in the SPL and the adjacent inferior parietal lobule (Hutchison et al., 2012). Two previous studies that applied online TMS to the right FEF have found enhanced contrast perception. Ruff et al. (2006) applied HF-rTMS while recording fMRI BOLD signals and found that it enhanced activity in parts of retinotopic maps V1-4 that map the visual periphery, and decreased activity centrally. This led them to predict that perceived contrast would be enhanced in the periphery relative to the central visual field by TMS to the FEF, and this prediction was confirmed psychophysically. Enhanced contrast detection was also found by Chanes et al. (2012) for single pulse TMS applied to the FEF with stimuli presented in the visual periphery (central stimuli were not tested). The low contrast targets in our experiment were also presented peripherally, cantered at 5 degrees of eccentricity, and we also found improved contrast discrimination. One interpretation of this finding is that early visual areas and contrast discrimination in our experiment were influenced indirectly by TMS via the strong connections between the SPL and the FEF, but equally it is possible that the effect was more direct. Further studies are required to address this question, and should also include psychophysical measurements of contrast detection and discrimination in the central visual field to find out whether, as for TMS to the FEF, the effect is confined to peripheral vision.

In conclusion, TMS to the SPL did not influence the precision of perceptual judgements of visual separation, and so the results failed to support our main hypothesis. This puts them at odds with the suggestive results from brain imaging, and one potential route for further exploration would be to perform an experiment that asks whether TMS to SPL can produce a bias in perceptual judgments of visual separation. We unexpectedly found that TMS to SPL increased the precision of peripheral contrast judgement, and because similar results have been obtained when TMS is delivered to the closely connected FEF this suggests that follow-up experiments should seek to map out the respective roles of the two areas in producing this effect, and how it is reflected in occipital retinotopic regions.
